# The alternative sigma factor SigB of *Corynebacterium glutamicum *modulates global gene expression during transition from exponential growth to stationary phase

**DOI:** 10.1186/1471-2164-8-4

**Published:** 2007-01-04

**Authors:** Christof Larisch, Diana Nakunst, Andrea T Hüser, Andreas Tauch, Jörn Kalinowski

**Affiliations:** 1 Institut für Genomforschung, Centrum für Biotechnologie, Universität Bielefeld, 33594 Bielefeld, Germany

## Abstract

**Background:**

*Corynebacterium glutamicum *is a gram-positive soil bacterium widely used for the industrial production of amino acids. There is great interest in the examination of the molecular mechanism of transcription control. One of these control mechanisms are sigma factors. *C. glutamicum *ATCC 13032 has seven putative sigma factor-encoding genes, including *sigA *and *sigB*. The *sigA *gene encodes the essential primary sigma factor of *C. glutamicum *and is responsible for promoter recognition of house-keeping genes. The *sigB *gene codes for the non-essential sigma factor SigB that has a proposed role in stress reponse.

**Results:**

The *sigB *gene expression was highest at transition between exponential growth and stationary phase, when the amount of *sigA *mRNA was already decreasing. Genome-wide transcription profiles of the wild-type and the *sigB *mutant were recorded by comparative DNA microarray hybridizations. The data indicated that the mRNA levels of 111 genes are significantly changed in the *sigB*-proficient strain during the transition phase, whereas the expression profile of the *sigB*-deficient strain showed only minor changes (26 genes). The genes that are higher expressed during transition phase only in the *sigB*-proficient strain mainly belong to the functional categories amino acid metabolism, carbon metabolism, stress defense, membrane processes, and phosphorus metabolism. The transcription start points of six of these genes were determined and the deduced promoter sequences turned out to be indistinguishable from that of the consensus promoter recognized by SigA. Real-time reverse transcription PCR assays revealed that the expression profiles of these genes during growth were similar to that of the *sigB *gene itself. In the *sigB *mutant, however, the transcription profiles resembled that of the *sigA *gene encoding the house-keeping sigma factor.

**Conclusion:**

During transition phase, the *sigB *gene showed an enhanced expression, while simultaneously the *sigA *mRNA decreased in abundance. This might cause a replacement of SigA by SigB at the RNA polymerase core enzyme and in turn results in increased expression of genes relevant for the transition and the stationary phase, either to cope with nutrient limitation or with the accompanying oxidative stress. The increased expression of genes encoding anti-oxidative or protection functions also prepares the cell for upcoming limitations and environmental stresses.

## Background

The RNA polymerase of prokaryotic organisms is composed of distinct subunits: β, β', ω, an α dimer, and a σ factor [1]. The sigma factor of the RNA polymerase confers specificity to the process of transcription initiation by recognition of specific promoter sequences of genes and operons [2]. Under normal growth conditions, bacteria use generally a RNA polymerase holoenzyme containing the principal sigma factor SigA. This sigma factor is essential for the transcription of house-keeping genes [3]. It is generally observed that under nutrient limitation or under a variety of physical and chemical stresses, additional sigma factors compete for the limited amount of RNA polymerase core enzyme. These sigma factors are non-essential for exponential growth and fall into different families. The first family comprises sigma factors of the non-essential sigma factors subgroups 2.1 from gram-negative bacteria, such as σ^S ^from *Escherichia coli*, and 2.3 comprising the σ^B ^factors from gram-positive Actinobacteria [4]. These sigma factors are similar to the primary sigma factor in the amino acid sequence of the DNA-binding region, which suggests that both groups of sigma factors recognize similar promoter sequences [5,6]. The other family comprises alternative sigma factors of the subgroup 3.3, such as σ^B ^of *Bacillus subtilis *and related gram-positive Firmicutes [7] which recognize promoters with a different consensus sequence [4].

In gram-negative bacteria like *E. coli *the non-essential sigma factor σ^S ^(RpoS) is strongly induced during entry into the stationary phase as well as under stress conditions and is essential for the expression of multiple stress resistance genes [8]. In *Mycobacterium tuberculosis*, the non-essential sigma factor σ^B ^behaves like σ^S ^of *E. coli *[9] since its transcription is induced during transition from exponential to stationary phase and under certain stress conditions. Therefore, it has been suggested that RpoS and mycobacterial SigB play similar roles in the general stress response of gram-negative and gram-positive bacteria [10]. In *B. subtilis*, the unrelated alternative sigma factor σ^B ^regulates the transcription of a large number of general stress operons, thereby contributing to the transcription of more than 200 genes involved in heat, acid, ethanol, salt, and oxidative stress resistance [11,12].

*Corynebacterium glutamicum *is a gram-positive non-pathogenic soil bacterium widely used for the production of amino acids. There is great interest in the examination of amino acid biosynthesis pathways and the molecular mechanism of transcription control [13,14]. One of these control mechanisms at the transcriptional level are sigma factors. Annotation of the genome sequence of *C. glutamicum *ATCC 13032 revealed the presence of seven putative sigma factor genes, including *sigA *and *sigB *[15,16]. The *sigA *gene encodes the essential primary sigma factor of *C. glutamicum *and is responsible for promoter recognition of house-keeping genes [17,18]. The nucleotide sequence of the -10 region of the SigA consensus promoter sequence is tgngnTA(c/t)aaTgg [19]. The *sigB *gene encoding the non-essential sigma factor SigB is transcribed during the exponential growth phase and transcript abundance ceases in stationary phase [20]. Halgasova et al. [21] showed that SigB is involved in the response to several environmental stresses, such as acids, ethanol, cold, and heat shock and that disruption of the *sigB *gene leads to a substantially diminished growth of the mutant in shake-flask cultures. In this study, we analyzed by DNA microarray hybridizations the role of SigB during the transition of *C. glutamicum *growth phases to get a detailed and genome-wide view on the modulation of gene expression.

## Results

### The growth behavior of both a sigB-deficient and sigB-proficient *C. glutamicum *strains was comparable under controlled cultivation conditions in glucose-limited batch fermentations

To investigate the physiological role of the sigma factor SigB in *C. glutamicum*, a *sigB *deletion mutant was constructed by gene replacement in *C. glutamicum *RES167, a restriction-deficient derivative of the wild-type strain ATCC 13032, and designated *C. glutamicum *CL1. The deletion introduced into the *sigB *gene was 771 bp in size and removed the coding sequence for 257 of the 331 amino acids, including the highly conserved protein regions 1, 2 and 3 of sigma factor proteins [22].

**Figure 1:**
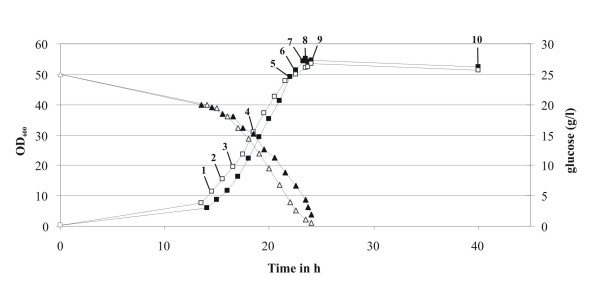
Growth and sugar consumption during batch-fermentation of *C. glutamicum *RES167 (black squares) and the *sigB *mutant *C. glutamicum *CL1 (open squares) in minimal medium MM1 at 30°C and pH 7. Growth was monitored by measuring the optical density (o.D.) at a wavelength of 600 nm. Black triangles symbolize the remaining glucose in cultures of *C. glutamicum *RES167, open triangles show the glucose concentration in cultures of the *sigB *mutant *C. glutamicum *CL1. The numbers correspond to different sampling points of *C. glutamicum *RES167 and *C. glutamicum *CL1 cells for further analyses.

A glucose-limited batch fermentation was chosen for cultivation of both the *sigB*-proficient strain *C. glutamicum *RES167 and the *sigB*-deficient strain *C. glutamicum *CL1. The cultures were grown at 30°C in a fermentor with a constant pO_2 _level of 30% and a pH of 7. Beside the impeller speed, carbon dioxide production was monitored online, and the remaining glucose was determined by off-line measurements. The time point of glucose exhaustion and the transition from exponential to stationary phase are precisely defined by an immediate drop in carbon dioxide production at glucose exhaustion (data not shown). Under these conditions, the growth profiles of both cultures (Fig. 1) were similar and also the maximal growth rates differed only slightly (RES167: 0.22 ± 0.03 h^-1^; CL1: 0.27 ± 0.02 h^-1^).

### The *C. glutamicum *sigB gene encoding an alternative sigma factor was preferentially transcribed during transition from exponential growth to stationary phase

**Figure 2:**
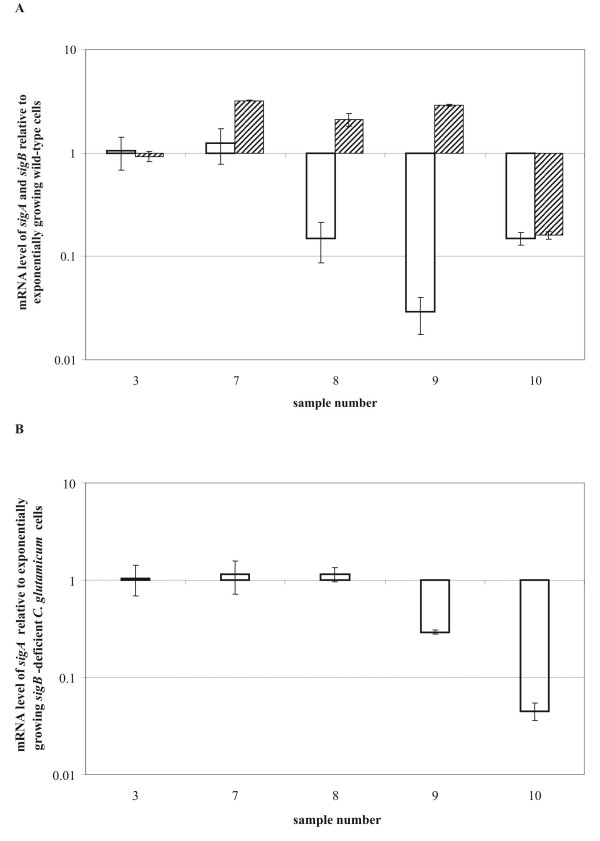
Relative levels of *sigA *and *sigB *mRNA in *C. glutamicum *RES167 at different phases of growth. **(A) **The bars show mRNA levels of *sigA *(solid bars) and *sigB *(speckled bars) calculated in relation to those determined at early exponential growth phase in the wild-type. The sample numbers correspond to the sampling points marked in Figure 1. **(B) **The bars show mRNA levels of the *sigA *gene in the *sigB*-deficient *C. glutamicum *strain CL1.

To determine whether the sigma factor gene expression is influenced by the growth phase of *C. glutamicum *cultures, transcription profiles of the *sigA *gene encoding the house-keeping sigma factor and of the *sigB *gene were recorded. Both strains were cultivated in the controlled environment of a fermentor minimizing the influence of environmental stresses and differences in growth rates between both strains. The transition phase was induced in a reproducible manner by limiting the carbon source glucose. Thus, *C. glutamicum *RES167 and CL1 cells were harvested during exponential growth phase (Fig. 1; sample number 1 to 6), during transition phase (sample number 7 and 8) and during the stationary growth phase (sample number 9 and 10). The sample numbers correspond to the different growth phases of *C. glutamicum *cells for all further analyses. The amounts of *sigA *and *sigB *transcripts were then determined by real-time RT-PCR. Figure 2A shows the relative amounts of *sigA *and *sigB *transcripts in *C. glutamicum *RES167 at different points of the growth profile in comparison to those determined at mid-exponential phase (time point 4 in Fig. 1).

The analysis indicated that *sigA *is almost constantly transcribed during the exponential growth phase. Such an expression pattern has been reported also for the main sigma factor σ^70 ^of *E. coli *[2]. At transition phase (sample number 8), a significant decline in abundance of *sigA *mRNA was observed in *C. glutamicum *RES167 (Fig. 2A). The *sigB *mRNA abundance was highest at the transition phase (sample numbers 7 and 8) and, unlike to that of *sigA*, remained at an increased level in early stationary phase (sample number 9). At later stages of the stationary phase the *sigB *mRNA level decreased (Fig. 2A). The mRNA levels of *sigA *were also determined for the *sigB*-deficient *C. glutamicum *mutant strain CL1 (Fig. 2B). Surprisingly, the expression of *sigA *remained at identical levels during exponential growth and transition phase (sample number 8), whereas a decreased expression was observed again in early and late stationary phase (sample numbers 9 and 10).

### Global gene expression of a sigB-proficient and a sigB-deficient *C. glutamicum *strain differed significantly during transition phase

The *sigB*-proficient strain RES167 and its derived *sigB*-deficient deletion mutant CL1 were used to identify genes that are transcribed under the control of SigB by microarray hybridization.

In total, three different microarray experiments were carried out. In the first experiment, the global gene expression of the *sigB*-proficient strain RES167 was compared to that of the *sigB*-deficient strain CL1 whereby both strains were harvested in the exponential growth phase (sample number 4). This experiment should clarify whether SigB plays a role in global gene expression in the exponential growth phase. In the second experiment, the global gene expression of the *sigB*-proficient strain RES167 was compared between the transition growth phase (sample number 8) and exponential growth phase (sample number 4). This experiment was designed to monitor the changes in the global gene expression of the *sigB*-proficient strain between the exponential and the transition growth phase. In the third experiment, the global gene expression of the *sigB*-deficient strain CL1 was compared between the same two growth phases, namely the exponential growth phase (sample number 4) and the transition growth phase (sample number 8). The third experiment had the same design as the second one with the only difference that the *sigB*-deficient strain was analyzed. Genes that were found differentially expressed in this experiment are apparently regulated independently of SigB.

For microarray analyses, *C. glutamicum *RES167 and CL1 cells were harvested during exponential phase (Fig. 1; sample number 4) or during transition phase (sample number 8), respectively. Total RNA samples were prepared from two independently grown cultures of each strain at the two time points and each RNA preparation was used in two hybridization assays, applying label-swapping. Therefore, differential gene transcription was determined by four DNA microarray hybridizations and a total of 16 gene replicates. Labeling of probes, array hybridization and data evaluation were carried out as described previously [23]. Normalization by the LOWESS function and *t*-test statistics were accomplished with the EMMA microarray data analysis software [24]. In all experiments, an *m*-value cut-off of ± 1.0, corresponding to relative expression changes equal or greater than 2, was applied.

In the first experiment (RES167/exp vs. CL1/exp), no gene was detected that delivered a significant change in gene expression (data not shown). This indicated that the absence of SigB did not result in differential gene expression during exponential growth phase and that the transcription of *sigB *in the exponential phase of the *C. glutamicum *RES167 strain has no effect during the exponential growth phase, too. This experiment allowed us to compare the results of the two following experiments directly.

**Figure 3:**
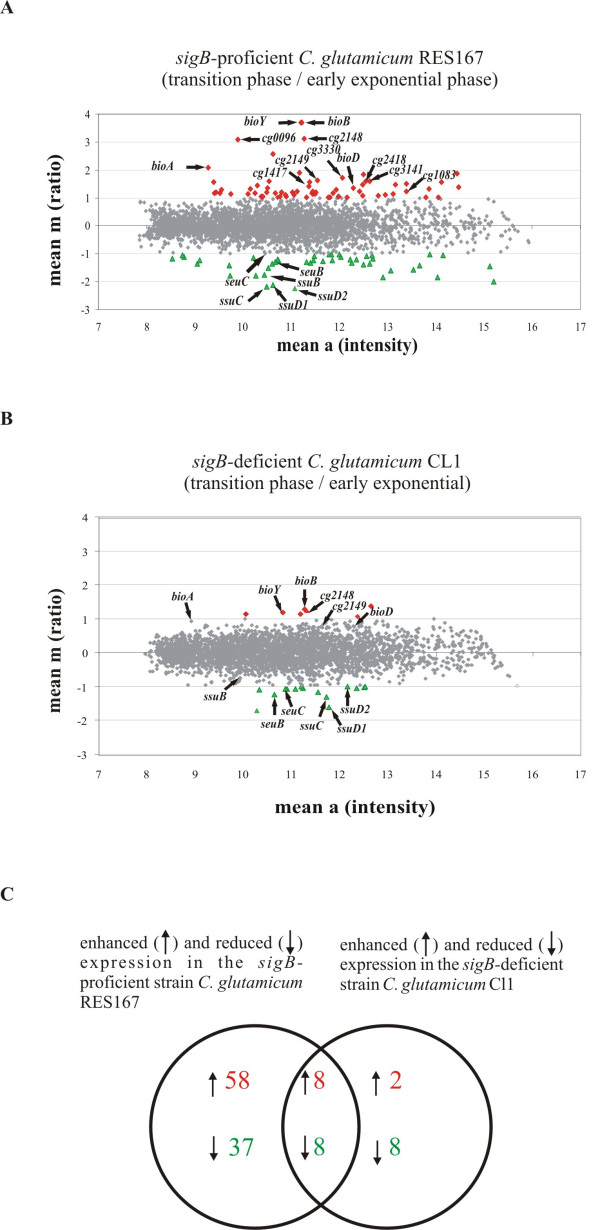
Identification of SigB-dependent genes by DNA microarray hybridization. Ratio/intensity (m/a) plots of DNA microarray hybridizations of *sigB*-proficient *C. glutamicum *RES167 **(A) **and *sigB*-deficient *C. glutamicum *CL1 **(B) **comparing genome-wide transcription at transition phase with that at early exponential phase. Diamonds represent genes with enhanced mRNA levels during transition phase; triangles represent genes with decreased mRNA levels. Each data point was calculated as the average of two biological and two technical replicates including dye-swapping. Selected genes, which were discussed in the text, are indicated **(C) **The diagram shows the numbers of genes with enhanced and reduced expression during transition phase in the *sigB*-proficient strain *C. glutamicum *RES167 (left), the *sigB*-deficient strain *C. glutamicum *CL1 (right) and in both strains (middle).

In the second experiment (RES167/trans vs. RES167/exp), a total number of 111 genes revealed differential expression, including 66 genes with significantly increased expression (e.g. *bioY*,*bioB*, *bioA *and *aroF*) and 45 genes with decreased expression in the transition phase (e.g. *seuC*, *seuB*, *ssuD2*, *ssuC*, *ssuD1 *and *ssuB*) (Fig. 3A). In the third experiment (CL1/trans vs. CL1/exp), 26 genes had a different expression level in transition phase, including 10 genes with increased expression (*bioB*, *bioY *and *phoD*) and 16 genes with decreased expression (e.g. *seuC*, *seuB*, *ssuD2*, *ssuC*, *ssuD1 *and *ssuB*) (Fig. 3B). This experiment demonstrates, that in the strain *C. glutamicum *CL1 fewer genes were differentially expressed during the transition growth phase.

These data sets enabled us to classify all genes according to their expression behavior in the latter two experiments (Fig. 3C): the first class comprises genes with altered mRNA levels at transition phase in *C. glutamicum *RES167, compared to those showing altered mRNA levels during transition phase in *C. glutamicum *CL1. In total, 58 genes were identified that revealed an enhanced transcription during transition phase and 37 genes had decreased mRNA levels at transition phase only in the presence of a functional *sigB *gene (Table 1). The genes of the first class can be considered as being transcribed with the help of SigB, whereas those of the second class require other explanations. They might either be influenced by transcriptional regulators that are expressed with the help of SigB or might be transcribed by another sigma factor and are therefore sensitive to the lowered amount of free RNA polymerase holoenzyme.

For defining the second class, genes differentially expressed in transition phase independent of SigB were identified (Table 2). A closer inspection of these genes revealed that they essentially belong to two different functional complexes. First, these were the biotin biosynthesis and transport genes *cg0095 *(*bioB*), *cg2147 *(*bioY*)-*cg2149*, *cg2885 *(*bioA*) *cg2886 *(*bioD*) which were all found stronger expressed in transition phase in the *sigB*-proficient and the *sigB*-deficient strain. This expression pattern indicates an additional biotin limitation in the fermentation, since biotin had to be added as a supplement due to an auxotrophy of *C. glutamicum *[25]. Second, the genes and operons involved in utilization of sulfonates and sulfonate esters as sulfur sources *cg1147 (ssuI)-cg1156 (ssuD2)*, *cg1376(ssuD1)-cg1379(ssuB) *[26], are all downregulated in transition growth phase. This can be interpreted as the reflection of a higher concentration of free sulfate or sulfite in the cell at transition growth phase since these substances are known to inactivate the transcriptional activator of these genes, SsuR [27].

A very small number of genes showed differential expression at transition phase only in the *sigB *mutant strain (data not shown). Since these genes display rather small ratios and their gene products have not been studied in *C. glutamicum*, they will not be discussed further.

### Classification of genes differentially transcribed only in the sigB-proficient *C. glutamicum *strain

The genes that display differential expression in the transition growth phase only in the presence of *sigB *were ordered into nine functional classes according to the annotation of their gene products (Table 1). The first class *Amino Acid Metabolism and Transport *comprises genes encoding proteolytic enzymes (*cg0998*, *cg1930*) and the uptake of peptides (*cg2884*) which are all found to be upregulated. In addition, genes encoding the first step of aromatic amino acid biosynthesis (*aroF*) and the final step in aromatic and branched-chain amino acid biosynthesis (*ilvE*) are upregulated. In contrast to this, the gene encoding the first step in proline biosynthesis (*proB*) and second second-last step in leucine biosynthesis (*leuB*) are found to be downregulated. The gene products of *leuB *and *ilvE *encode consecutive reactions in leucine biosynthesis having the common intermediates 2-oxo-4-methyl-3-carboxy-pentanoate and 4-methyl-2-oxopentanoate which is a spontaneously occurring decarboxylation product.

It is interesting to note that only few genes involved in the second functional class *Carbon Metabolism and Transport *were upregulated depending of *sigB*. Among these genes is *cg1479 *(*glgP1*) which encodes a putative glucan phosphorylase responsible for the mobilization of carbon storage reserves such as glycogen. Other genes involved in carbon metabolism, like *cg0756 *(*cstA*), which encodes a putative carbon starvation protein, *cg1791 *(*gap*) and *cg1790 *(*pgk*), which encode glyceraldehyde-3-phosphate dehydrogenase and phosphoglycerate kinase taking part in glycolysis, respectively, are repressed during transition phase. Both genes are located together in an operon of the order *gap-pgk-tpi-ppc *further encoding triose-phosphate isomerase and PEP carboxylase, respectively [28]. In addition, further genes encoding putative enzymes involved in carbon metabolism or in the uptake of sugars (*cg0699*, *cg2705*/*amyE*-*cg2704*) were found to be repressed in the *sigB*-proficient strain.

In the third class *Stress Defense Mechanisms *the picture is the opposite. Here a considerably high number of putative detoxification genes encoding glyoxlase (*cg1073*), methionine-R-sulfoxide reductase (*cg2078*) or the nitric oxide-detoxification flavohemoprotein (*hmp*) were found to be upregulated. In contrast to this, only the genes encoding a putative universal stress protein (*uspA2*) and the chaperone Hsp70 (*dnaK*) were downregulated.

A high number of genes coding for proteins involved in *Membrane Processes *are differentially regulated in transition phase. Here the picture is not very clear but the genes involved in processes like metal uptake (*cg0467*, *cg1623*, *cg2676*) or cell wall lipid carrier biosynthesis (*uppS1*) were found to be upregulated whereas those encoding other membrane proteins or the two porins (*porB*, *porC*) were downregulated. It is interesting to note that three of the membrane proteins containing the energy-sensing CBS-domain [29] were downregulated, too.

The class *Phosporus Metabolism and Regulation *largely comprises genes found to be upregulated. These genes apparently encode functions involved in phospholipid metabolism (*cg1718*, *cg3194*) and regulation of phosphate uptake or the phosphate starvation response (*phoU, phoR*). In *E. coli *the PhoU protein senses the concentration of intracellular inorganic phosphate and is a negative regulator of organophosphate uptake and polyphosphate formation [30]. The *C. glutamicum phoR *gene encodes the transcriptional regulator of the recently discovered two-component regulatory system PhoRS [31] and is supposed to activate transcription of genes necessary for survival under low phosphorus concentrations. However, none of the genes differentially regulated in dependence on *sigB *is a member of the regulon reacting on the intracellular inorganic phosphate level [32].

The sixth class *Regulatory Processes *comprises four genes with induced transcription. These encode another two-component sensor/regulator system (*cgtS10*, *cgtR10*), a TetR-type and a λ repressor-like transcriptional regulator. Unfortunately, it is not known which signals trigger these regulators or which genes are regulated by them. They might be among those that are differentially regulated only in the *sigB*-proficient strain.

The class *Transcription and Translation *includes three genes, which are all downregulated. The *rplJ *and *rplL *genes lie together in an operon and encode the ribosomal protein subunits L10 and L7/L12. The same growth phase-dependent transcription of the *rplJL *operon showing a decrease at transition phase was already shown for another actinomycete, *Streptomyces coelicolor *[33]. The third gene in this category is *sigA *encoding the essential house-keeping sigma factor SigA. In concordance with the transcription profiles established by RT-PCR before, the *sigA *transcript was found to be less abundant in transition growth phase when a functional *sigB *gene is present. This is an indication that the apparent downregulation of some genes during transition phase is an indirect consequence of the higher level of *sigB *expression.

In the class *Vitamins/Cofactors Biosynthesis and Transport*, three genes involved in different cofactor synthesis pathways are placed. The gene *coaA *encodes pantothenate kinase, catalyzing the first step of coenzyme A biosynthesis [34] and being upregulated at transition phase. Another gene involved in pyridoxine biosynthesis was upregulated (*cg0999*), whereas a third gene involved in molybdenum cofactor biosynthesis was found to be downregulated (*cg0899*). Here no consistent regulatory pattern was apparent.

The class *Function Unknown *comprises 25 genes the products of which are present also in other bacteria (conserved hypothetical proteins) but have only an ill-defined or entirely unknown function. The larger fraction of these genes or operons was found to be upregulated in the presence of *sigB*.

### Mapping of promoters in front of genes showing an elevated expression in the sigB-proficient *C. glutamicum *strain at transition phase

**Figure 4:**
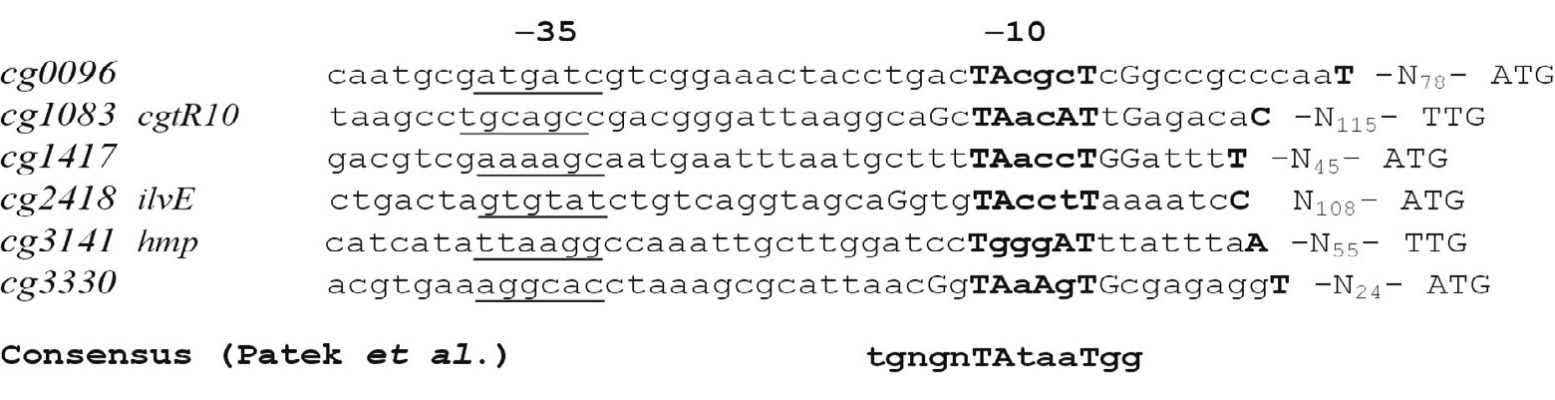
Promoter sequences of *C. glutamicum *genes showing elevated expression at the onset of stationary phase. The transcription start sites mapped by RACE PCR assays are shown in boldfaced, capital letters. The numbers indicates the position of the transcription start site relative to the translational start codon. The deduced -10 sequences are shown in bold, the -35 elements are underlined. Nucleotides that match the proposed consensus for the -10 element of *C. glutamicum *are symbolized by capital letters. The consensus sequence of the -10 element of the *C. glutamicum *sigma factor SigA [tgngnTAtaaTgg] is shown for comparison [19].

RACE-PCR assays were performed to determine the promoter sequences of the six genes/operons *cg0096/cg0097, cg1083 (cgtS10)/cg1084 (cgtR10), cg1417, cg2418 (ilvE), cg3141 (hmp)*, and *cg3330*. These genes were selected because their mRNA abundances showed high ratios during the transition phase only in the presence of SigB. The RACE-PCR was performed by using total RNA of *C. glutamicum *RES167 harvested during transition phase, and the transcriptional start sites were determined by comparison to the whole genome sequence [16]. The results of promoter mapping are shown in Figure 4. The deduced -35 and -10 promoter sequences of the investigated genes were indistinguishable from the consensus promoter sequence of *C. glutamicum *[19].

### Expression profiles of genes showing an elevated expression in the sigB-proficient *C. glutamicum *strain at transition phase

**Figure 5:**
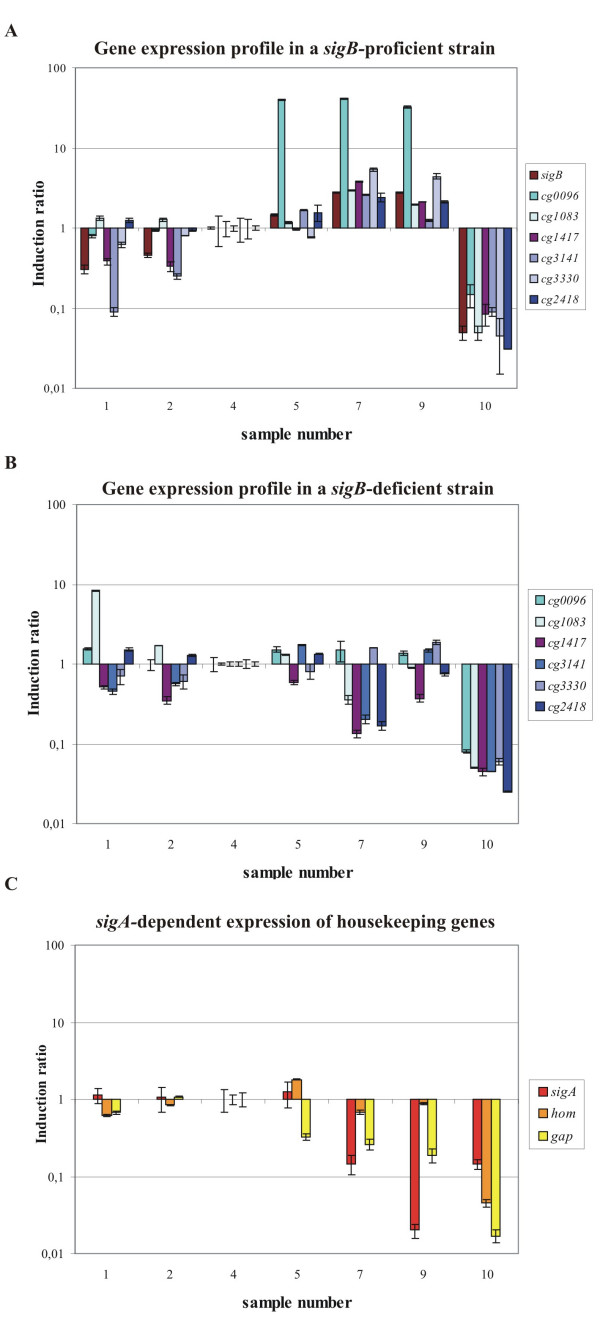
Real-time RT-PCR analysis of relative transcription levels of *sigB, cg0096, cg1083, cg1417, cg3141, cg3330, cg2418, sigA, hom*, and *gap *during growth. The sample numbers refer to the growth curves shown in Figure 1. Transcription levels were calculated relative to sample number 4 (early exponential phase). The diagrams show gene expression in the *sigB*-proficient strain *C. glutamicum *RES167 **(A) **and in the *sigB*-deficient strain *C. glutamicum *CL1 **(B)**. Diagram **(C) **shows relative expression levels of known SigA-dependent genes of *C. glutamicum *RES167.

Furthermore, transcription profiles of *sigB, cg0096, cg1083, cg1417, cg2418, cg3141, cg3330, sigA, hom*, and *gap *were determined by real-time RT-PCR during growth of *C. glutamicum *RES167. The house-keeping genes *sigA*, *hom *(encoding homoserine dehydrogenase) and *gap *(encoding glyceraldehyde-3-phosphate dehydrogenase) served as controls since they are known to be transcribed by SigA [19]. Transcription profiles of *cg0096, cg1083, cg1417, cg2418, cg3141*, and *cg3330 *in the *sigB*-proficient background of *C. glutamicum *RES167 were very similar to that of *sigB *during different growth phases (Fig. 5A). In the *sigB *mutant *C. glutamicum *CL1, these profiles changed and were apparently different to those of *C. glutamicum *RES167, especially during transition phase (Fig. 5B). However, the expression profiles of the selected genes of *C. glutamicum *CL1 became very similar to those of the SigA-transcribed genes *sigA*, *hom *and *gap *of *C. glutamicum *RES167 (Fig. 5C).

## Discussion

### SigB is involved in regulation of transition from exponential growth to stationary phase

Our data revealed that SigB is not only necessary for stress response, but also for growth phase-dependent gene regulation. After batch fermentation of the *sigB*-proficient and *sigB*-deficient *C. glutamicum *strain, the generation time of both were rather similar. These results were in contrast to those of Halgasova et al. who recorded a severe growth defect of a *sigB*-disrupted *C. glutamicum *mutant [21]. The main difference between both experimental approaches is apparently the method of cultivation. We employed a fermentor for the cultivation of *C. glutamicum *strains and thus avoided specific stress conditions that might result in growth deficiency of a *sigB *mutant as observed during cultivation in shake-flasks without aeration and pH control [21]. This notion is supported by the previous observation that expression of the *sigB *gene increased after supplying a number of environmental stress conditions to *C. glutamicum *cultures. However, it remains to be investigated whether occasional stress in shaking-flask cultures such as shifting pH or limited dissolved oxygen is a trigger for SigB activity. Furthermore, the transcription analysis of *sigA *and *sigB *clarified the different dependency of expression during different growth phases. Real-time RT-PCR analysis showed that transcription of the *sigB *gene was significantly increased when *C. glutamicum *entered the transition growth phase. This finding supports the assumption that SigB is the alternative sigma factor of *C. glutamicum *and is not only involved in stress adaptation but also in growth phase-dependent gene expression [17,18]. Moreover, the transcription profile of the *C. glutamicum sigB *gene is very similar to that of the orthologous *sigB *gene of *M. tuberculosis *[9] and to the expression pattern of the *rpoS *gene encoding the alternative sigma factor σ^S ^of *E. coli *[35]. In all cases, transcription of the gene was maximal during transition from exponential to stationary phase as well as under certain environmental stress conditions [2,36]. For *C. glutamicum*, Oguiza et al. analyzed the abundance of *sigA *and *sigB *transcripts during growth in complex medium by Northern hybridization and found out that both transcripts were abundant during exponential growth phase and abundance decreased simultaneously in stationary phase. During early exponential phase, the *sigA *transcript was more abundant than the *sigB *transcript [20].

### Transition from exponential to stationary growth phase changed the transcription profile of *C. glutamicum*

Comparative DNA microarray analyses between the *sigB*-proficient and *sigB*-deficient *C. glutamicum *strain delivered a genome-wide view on relative transcript abundances during transition phase. Quite a large number of genes identified here falls into three functional classes. They encode either carbon metabolism or transport, stress defence and membrane processes. Weber *et al*. identified several hundred genes as to be controlled by the non-essential sigma factor σ^S ^in *E. coli *which is also higher expressed during transition phase. A large number of the respective proteins with known or predicted functions fall into similar classes as in our study. These are protein-processing reactions, stress-defence mechanisms, membrane processes and regulatory functions [8]. It can be assumed that *E. coli *SigS as well as *C. glutamicum *SigB have similar functions as main regulators of cellular functions at suboptimal growth, e.g. the scavenging of various nutrients and increased resistance against various toxic compounds. The genes that are regulated by the putative SigB-ortholog in *S. coelicolor *also belong to similar functional complexes. Especially, a large number of genes encoding membrane-associated, secreted and cell-wall-related proteins are transcribed with the help of SigB, suggesting the involvement in protection against oxidative damage and osmotic stress [37].

Reduced transcription of genes under conditions of an enhanced expression of *sigB *may occur through a competition for the core polymerase between SigA and SigB. Because of this competition, the expression of the *sigA *gene also decreases in the transition phase since the *sigA *gene is apparently transcribed with the help of SigA [19]. The genes negatively affected by the presence of SigB might therefore be transcribed predominantly with the help of SigA in *C. glutamicum*. Studies in *E. coli *demonstrated that the affinity of σ^S ^for the core RNA polymerase is lower than that of σ^70 ^[38,39], and since the amount of RNA polymerase is limiting, there is a competition between both sigma factors for binding to the core enzyme during transition phase [40]. The competitiveness of a given sigma factor for the core RNA polymerase is determined by its abundance in the cell and its relative affinity for the RNA polymerase [41]. Therefore, a concomitant decrease in abundance of SigA and an increase of SigB in *C. glutamicum *during transition phase should support the interaction of SigB with the RNA polymerase core enzyme and should lead to a high level expression of certain genes, fulfilling a vital role in this phase and later stages of growth.

## Conclusion

In this study, we demonstrated that SigB is involved in gene regulation at the transition from exponential to stationary growth phase. During transition phase, the *sigB *gene showed an enhanced expression, while simultaneously *sigA *mRNA decreased in abundance. This might cause a replacement of SigA by SigB at the RNA polymerase core enzyme and in turn results in increased expression of genes relevant for transition phase of growth, either to cope with nutrient limitation or with the accompanying oxidative stress. The increased expression of genes encoding anti-oxidative or protection functions also prepares the cell for upcoming limitations and environmental stresses. In this functional role the *C. glutamicum *SigB is similar to RpoS of *E. coli*. However, this study has only addressed a single functionality of SigB and other physiological roles, especially with respect to certain environmental stresses might exist. These functional complexes as well as the potential regulation of the *sigB *gene and its encoded protein by other transcriptional regulators or by anti-sigma factors are interesting subjects of future studies.

## Methods

### Bacterial strains and growth conditions

*E. coli *was routinely grown at 37°C in Luria-Bertani medium [42] supplemented with 2 g/l glucose (LBG). *C. glutamicum *strains were grown at 30°C in minimal medium MM1 (MMYE without yeast extract) [43]. For batch cultures, 100 ml of stationary shake-flask cultures of *C. glutamicum *was used to inoculate a 7-l fermentor (MBB, Büchi, Switzerland) containing 5 l minimal medium MM1. The cultures were grown at 30°C with a pO_2 _level of 30%. The pH set point was 7, regulated with 2 M NaOH and 10% (w/v) H_3_PO_4_. Glucose was limited (25 g/l) to induce the stationary phase by glucose exhaustion.

### DNA and PCR techniques

*E. coli *DH5αMCR [44] was used for cloning experiments. Vector DNA was prepared from *E. coli *cells by alkaline lysis using the QIAprep Spin Miniprep Kit (Qiagen, Hilden, Germany). DNA restriction fragments required for cloning were purified from agarose gels by means of the QIAEX II Gel Extraction Kit (Qiagen). All recombinant DNA techniques followed standard procedures [42]. *E. coli *and *C. glutamicum *cells were transformed by electroporation [45,46]. Chromosomal DNA of *C. glutamicum *was prepared as described earlier [47].

PCR experiments were carried out with a PTC-100 thermocycler from MJ Research (Watertown, MA) and *Pfu *DNA polymerase. Initial denaturation was conducted at 94°C for 2 min followed by denaturation for 30 s, annealing for 30 s at a primer-dependent temperature, and extension at 72°C for 45 s. This cycle was repeated 30 times, followed by a final extension step at 72°C for 3 min. PCR products were purified by using the QIAquick PCR Purification Kit (Qiagen). Cloning of PCR products was performed in *E. coli *TOP10 by means of the Zero Blunt TOPO PCR Cloning Kit (Invitrogen, Karlsruhe, Germany).

### Construction of the sigB deletion mutant *C. glutamicum *CL1

A defined chromosomal deletion within the *sigB *gene of *C. glutamicum *RES167 [46] was constructed with the pK18 *mobsacB *vector system which helps to identify an allelic exchange by homologous recombination [48]. The respective plasmid (pCL1) was constructed by the gene SOEing technique [49]. Gene replacement in the chromosome of *C. glutamicum *RES167 was verified by PCR experiments.

### Total RNA isolation from *C. glutamicum *cells for DNA microarray hybridization

*C. glutamicum *cells were harvested during exponential, transition and stationary phase, as described previously [23]. Isolation of RNA was carried out by means of the RNeasy Mini Kit (Qiagen) following the manufacturer's instructions. The RNase-Free DNase set (Qiagen) was applied for on-column removal of DNA. A second DNase I digestion was performed by using the DNase I Kit (Sigma-Aldrich, Taufkirchen, Germany).

The *C. glutamicum *DNA microarray used in this study was developed by Hüser et al. [23]. Synthesis and labeling of cDNA as well as DNA microarray hybridization, signal detection and data analysis followed protocols described previously [23]. Monitoring of gene expression was performed with two biological replicates. Technical replicates were analyzed by using label-swapping, resulting in a total number of four measurements.

### Real-time RT-PCR assays

RT-PCR experiments were performed with the LightCycler instrument (Roche Diagnostics), using the QuantiTect SYBR Green RT-PCR Kit (Qiagen). Analyses were carried out with 1 μg of total RNA as template and the following cycler program: reverse transcription for 30 min at 45°C, initial activation for 15 min at 95°C and 3-step-cycling with denaturation for 10 s at 94°C, annealing for 40 s at 56°C and extension for 180 s at 68°C. Differences in gene expression were determined by comparing the crossing points of two samples measured in eight replicates. The crossing points were calculated by the LightCycler software version 3 (Roche Diagnostics).

### RACE-PCR assay for the identification of transcriptional start sites

Total RNA of *C. glutamicum *RES167 grown in MM1 medium was used for the determination of transcriptional start sites by means of the 5' RACE Kit (Roche Diagnostics). RACE PCR was carried out as recommended by the supplier, using 2 μg of total RNA. Resulting PCR products were ligated into the vector pCR2.1 by applying the TOPO TA cloning system and chemically competent *E. coli *TOP10 cells (Invitrogen). Sequencing of RACE products was carried out by IIT Biotech (Bielefeld, Germany).

## Authors' contributions

CL carried out the experimental work and drafted the manuscript. DN participated during mutant construction. ATH provided the DNA microarray. AT participated in data evaluation. JK conceived the study and participated in writing. All authors read and approved the final manuscript.

## Figures and Tables

**Table 1:** Genes with significantly altered expression at transition phase only in the *sigB*-proficient *C. glutamicum *strain (RES167).

CDS	Gene name	Predicted function	m-value
AMINO ACID METABOLISM AND PROTEOLYSIS			
*cg0998*		trypsin-like serine protease	1.57
*cg1129*	*aroF*	phospho-2-dehydro-3-deoxyheptanoate aldolase	1.87
*cg1783*	*soxA'*	glycine/D-amino acid oxidase – fragment	1.16
*cg1930*		secreted trypsine-like serine protease	1.29
*cg2418*	*ilvE*	branched-chain amino acid aminotransferase	1.48
*cg2884*		dipeptide/tripeptide permease	1.21
*cg1453*	*leuB*	3-isopropylmalate dehydrogenase	-1.04
*cg2588*	*proB*	glutamate 5-kinase protein	-1.43
CARBON METABOLISM AND TRANSPORT			
*cg1479*	*glgP1*	glycogen phosphorylase	1.17
*cg2796*		MmgE/PrpD family protein	1.19
*cg0699*	*guaB2*	inositol-monophosphate dehydrogenase, CBS domain	-1.07
*cg0756*	*cstA*	putative carbon starvation protein A	-1.61
*cg0812*	*dtsR1*	acetyl/propionyl-CoA carboxylase beta chain	-1.24
*cg1790*	*pgk*	phosphoglycerate kinase	-1.06
*cg1791*	*gap*	glyceraldehyde-3-phosphate dehydrogenase	-2.01
*cg2704*		sugar ABC-transporter, permease component	-1.38
*cg2705*	*amyE*	sugar ABC-transporter, binding protein	-1.42
*cg3096*		aldehyde dehydrogenase	-1.17
*cg3219*	*ldh*	L-lactate dehydrogenase	-1.10
*cg3380*		short-chain dehydrogenase	-1.06
STRESS DEFENSE MECHANISMS			
*cg1028*		restriction-modification system, methylase	1.04
*cg0291*		intradiol ring-cleavage dioxygenase	1.90
*cg1073*		glyoxylase	1.06
*cg2078*		methionine-R-sulfoxide reductase	1.09
*cg2999*		FAD-dependent oxidoreductase	1.12
*cg3141*	*hmp*	flavohemoprotein involved in NO detoxification	1.61
*cg1595*	*uspA2*	universal stress protein UspA	-1.11
*cg3100*	*dnaK*	heat shock protein Hsp70	-1.58
MEMBRANE PROCESSES			
*cg0465*		conserved hypothetical membrane protein	1.21
*cg0467*		cobalamin/Fe^3+^-siderophores transport system	1.22
*cg1082*		putative membrane protein	1.39
*cg1130*	*uppS1*	undecaprenyl pyrophosphate synthetase	1.50
*cg1344*	*narG*	nitrate reductase 2, alpha subunit	1.01
*cg1345*	*narK*	nitrate/nitrite transporter	1.36
*cg1622*		ABC-type multidrug/protein/lipid transport system	1.06
*cg1623*		divalent heavy-metal cations transporter	1.18
*cg2215*		membrane protein	1.25
*cg2377*	*mraW*	S-adenosylmethionine-dependent methyltransferase	1.03
*cg2425*		permease	1.01
*cg2440*		permease	1.23
*cg2676*		ABC-type peptide/nickel transport system	1.18
*cg2810*		Na^+^/H^+^-dicarboxylate symporter family	1.43
*cg3337*		putative membrane protein	1.18
*cg3338*		putative membrane protein	1.04
*cg1108*	*porC*	putative porin precursor	-1.25
*cg1109*	*porB*	anion-specific porin precursor	-1.84
*cg1349*		membrane protein, CBS domain	-1.79
*cg1639*		membrane protein, CBS domain	-1.23
*cg1640*		membrane protein, CBS domain	-1.52
*cg2467*		ABC-transporter ATP-binding protein	-1.33
*cg2468*		ABC-transporter permease component	-1.26
*cg2511*		putative membrane protein	-1.80
*cg3175*		membrane protein	-1.36
*cg3195*		flavin-containing monooxygenase involved in K^+ ^transport	-1.16
PHOSPHORUS METABOLISM AND REGULATION			
*cg0866*		phosphoribosyl transferase	1.02
*cg1718*		phospholipid-binding protein	1.02
*cg2842*	*phoU*	phosphate uptake regulator	1.31
*cg2888*	*phoR*	phosphate starvation two component response regulator	1.11
*cg3194*		membrane-associated phosphoesterase	1.06
*cg2513*	*phoH2*	phosphate starvation-inducible protein	-1.22
REGULATORY PROCESSES			
*cg1083*	*cgtS10*	probable two component sensor kinase	1.23
*cg1084*	*cgtR10*	two component response regulator	1.47
*cg2614*		bacterial regulatory proteins, TetR family	1.18
*cg3230*		transcriptional regulator, λ repressor-like	1.13
TRANSCRIPTION AND TRANSLATION			
*cg0572*	*rplJ*	50S ribosomal protein L10	-1.43
*cg0573*	*rplL*	50S ribosomal subunit protein L7/L12	-1.37
*cg2092*	*sigA*	RNA polymerase sigma 70 factor	-1.03
VITAMINS/COFACTORS BIOSYNTHESIS AND TRANSPORT			
*cg0999*		molybdenum cofactor biosynthesis protein	1.02
*cg1132*	*coaA*	pantothenate kinase	1.11
*cg0899*		glutamine amidotransferase/pyridoxine biosynthesis	-1.29
FUNCTION UNKNOWN			
*cg0096*		conserved hypothetical protein	3.08
*cg0097*		conserved hypothetical protein	2.57
*cg0753*		secreted protein	1.04
*cg0806*		conserved hypothetical protein	1.20
*cg0935*		conserved hypothetical protein	1.28
*cg1091*		hypothetical protein	1.85
*cg1131*		conserved hypothetical protein	1.59
*cg1286*		conserved hypothetical protein	1.55
*cg1304*		secreted protein	1.58
*cg1417*		GCN5-related N-acetyltransferase	1.41
*cg2057*		secreted protein	1.35
*cg2105*		conserved hypothetical protein	1.14
*cg2797*		conserved hypothetical protein	1.21
*cg2850*		conserved hypothetical protein	1.14
*cg3022*		conserved hypothetical protein	1.15
*cg3329*		conserved hypothetical protein	1.09
*cg3330*		putative secreted protein	1.71
*cg0177*		hypothetical protein	-1.21
*cg0757*		conserved hypothetical protein	-1.20
*cg0838*		DEAD-box helicase	-1.05
*cg0892*		conserved hypothetical protein	-1.03
*cg1013*		hypothetical protein	-1.30
*cg1167*		putative secreted protein	-1.14
*cg1911*		putative secreted protein	-1.07
*cg2464*		conserved hypothetical protein	-1.32

**Table 2:** Genes with significantly altered expression at transition phase in *sigB*-proficient *C. glutamicum *strain (RES167) and its derived *sigB*-deficient mutant (CL1).

CDS	Gene name	Predicted function	m-value RES167	m-value CL1
BIOTIN BIOSYNTHESIS AND TRANSPORT				
*cg0095*	*bioB*	biotin synthase	3.69	1.55
*cg2147*	*bioY*	membrane protein, BioY family	3.70	1.51
*cg2148*		ABC transporter, ATP-binding protein	3.11	1.51
*cg2149*		permease (cobalt permease subfamily)	1.63	0.75*
*cg2885*	*bioA*	adenosylmethionine-8-amino-7-oxononanoate aminotransferase	2.09	0.91*
*cg2886*	*bioD*	dethiobiotin synthetase protein	1.32	0.76*
*cg1227*		ABC-type cobalt transport system	1.58	1.48
FUNCTION UNKNOWN				
*cg0378*		putative phage-associated protein	1.14	1.61
SULFONATE (ESTER) METABOLISM AND TRANSPORT				
*cg1147*	*ssuI*	reductase involved in sulfonate degradation	-1.25	-1.70
*cg1152*	*seuB*	sulfonate ester monoxygenase	-1.35	-1.25
*cg1153*	*seuC*	sulfonate ester monooxygenase	-0.99*	-1.07
*cg1156*	*ssuD2*	alkanesulfonate monooxygenase	-2.23	-1.00
*cg1376*	*ssuD1*	alkanesulfonate monooxygenase	-2.11	-1.61
*cg1377*	*ssuC*	aliphatic sulfonates ABC transporter	-2.17	-1.32
*cg1379*	*ssuB*	aliphatic sulfonates ABC transporter	-1.75	-0.74*
CELL DIVISION				
*cg2378*	*mraZ*	MraZ protein	-1.02	-1.16

* m-values below the set cut-off are indicated in the case of proven or predicted operons
